# Supplementary material for: Allelic Variations in Phenology Genes of Eastern U.S. Soft Winter and Korean Winter Wheat and Their Associations with Heading Date
Source: Plants (Basel). 2022 Nov 15;11(22):3116. doi: 10.3390/plants11223116 (PMC9693561; doi:10.3390/plants11223116)
Supplement: Supplementary file 1 [file plants-11-03116-s001.zip › plants-1979392-supplementary.pdf]

plants

Supplementary Materials for

Article

Allelic Variations in Phenology Genes of Eastern U.S. Soft Winter and Korean Winter Wheat and Their Associations with Heading Date

Fengyun Ma [1,2], Gina Brown-Guedira [3], Moonseok Kang [4] and Byung-Kee Baik [1,*]

1 United States Department of Agriculture (USDA), Agricultural Research Service (ARS)-CSWQRU, Soft Wheat Quality Laboratory, 1680 Madison Avenue, Wooster, OH 44691, USA
2 Department of Horticulture and Crop Science, The Ohio State University, 1680 Madison Avenue, Wooster, OH 44691, USA
3 United States Department of Agriculture (USDA), Agricultural Research Service (ARS), Eastern Regional Small Grains Genotyping Laboratory, Raleigh, NC 27695, USA
4 Rural Development Administration, National Institute of Crop Science, Suwon, Gyeonggi 16429, Korea
* Correspondence: byungkee.baik@usda.gov

Supplemental Table S1 Phenology gene profiles and heading dates of 149 eastern U.S. soft winter wheat genotypes grown in Wooster, Ohio, for four years and 32 Korean winter wheat varieties grown in Wooster, Ohio, for two years.

The genotyped loci include vernalization (*Vrn*) genes where '*w*' indicates the winter allele, photoperiod (*Ppd*) genes where alleles are '*a*' insensitive and '*b*' sensitive, earliness per se (*Eps*) genes, reduced-height (*Rht*) genes where alleles are '*a*' tall and '*b*' semi-dwarf. Variation in winter alleles at vernalization loci include copy number variation (CNV) for *vrn-A1*, and Neuse-type ('*N*') or AGS2000-type ('*A*') alleles for *vrn-B1*. Missing heading data are indicated with a dash (-).

		Vrn loci					*Ppd* loci			*Eps* loci		*Rht* loci		Heading date (day of the year)			
Sample Name	Class	*Vrn-A1*	*vrn-A1* CNV	*Vrn-B1*	*vrn-B1*	*vrn-D3*	*A1*	*B1*	*D1*	*TaELF3-B1*	*TaELF3-D1*	*B1*	*D1*	2015	2016	2017	2018
U.S. soft winter wheat																	
Ackley	SRW	*w*	*>2*	*w*	*N*	*a*	*b*	*a-CS*	*b*	*a*	*b*	*b*	*a*	-	140	129	140
AG2581	SRW	*w*	*>2*	*w*	*N*	*b*	*a.1*	*null*	*b*	*a*	*a*	*b*	*a*	142	140	133	142
AG99751	SRW	*w*	*>2*	*w*	*N*	*a*	*b*	*a-CS*	*a/b het*	*a*	*b*	*b*	*a*	144	140	135	144
AgriMAXX 462	SRW	*w*	*>2*	*w*	*N*	*b*	*b*	*a-S64*	*a*	*a*	*a*	*a*	*b*	144	140	134	145
AGS 2056	SRW	*w*	*>2*	*w*	*N*	*b*	*b*	*b*	*a*	*a*	*a*	*b*	*a*	140	140	131	145
AGS 2060	SRW	*w*	*>2*	*w*	*A*	*b*	*b*	*a-CS*	*b-Nor*	*a*	*a*	*a*	*a*	144	140	135	147
Ambassador	SWW	*w*	*>2*	*w*	*N*	*b*	*b*	*b*	*a*	*a*	*b*	*a*	*b*	144	141	135	145
B06*0686	SRW	*w*	*2/>2 het*	*w*	*N*	*a*	*a.1*	*b*	*a/b het*	*a*	*het*	*het*	*het*	139	140	131	142
B08-91993	SRW	*w*	*>2*	*w*	*N*	*b*	*b*	*b*	*a*	*a*	*a*	*a*	*b*	140	140	133	143
B09-2950	SRW	*w*	*>2*	*w*	*N*	*a*	*a.1*	*a-CS*	*b*	*a*	*a*	*b*	*a*	141	140	132	145
Beck 113	SRW	*w*	*>2*	*w*	*N*	*b*	*b*	*b*	*a*	*a*	*a*	*a*	*b*	142	140	131	141
Beck 114	SRW	*w*	*>2*	*w*	*N*	*b*	*a.1*	*b*	*b*	*a*	*a*	*b*	*a*	-	139	132	143
Beck 120	SRW	*w*	*>2*	*w*	*N*	*b*	*b*	*b*	*a*	*a*	*a*	*b*	*a*	140	140	131	144
Beck 123	SRW	*w*	*>2*	*w*	*N*	*b*	*a.1*	*b*	*a*	*b*	*a*	*b*	*a*	-	140	132	144
Beck 125	SRW	*w*	*>2*	*w*	*N*	*b*	*b*	*null*	*a*	*a*	*a*	*b*	*a*	143	140	134	146
Beck 128	SRW	*w*	*>2*	*w*	*N*	*b*	*b*	*b*	*a*	*a*	*a*	*b*	*a*	-	140	135	145
Beck 129	SRW	*w*	*>2*	*w*	*N*	*b*	*b*	*b*	*a*	*a*	*a*	*a*	*b*	145	140	135	146

Supplemental Table S1 Continued

		Vrn loci					*Ppd* loci			*Eps* loci		*Rht* loci		Heading date (day of the year)			
Sample Name	Class	*Vrn -A1*	*vrn-A1* CNV	*Vrn-B1*	*vrn-B1*	*vrn-D3*	*A1*	*B1*	*D1*	*TaELF 3-B1*	*TaELF 3-D1*	*B1*	*D1*	2015	2016	2017	2018
Beck 88	SRW	*w*	*>2*	*w*	*N*	*a*	*a.1*	*b*	*b*	*a*	*het*	*b*	*a*	139	140	129	139
Bess	SRW	*w*	*>2*	*w*	*N*	*a*	*a.1*	*b*	*b*	*a*	*b*	*b*	*a*	142	138	134	144
Branson	SRW	*w*	*>2*	*w*	*N*	*b*	*a.1*	*b*	*a*	*a*	*het*	*b*	*a*	142	140	130	141
Bromfield	SRW	*w*	*>2*	*w*	*N*	*b*	*a.1*	*b*	*b*	*a*	*a*	*b*	*a*	145	142	136	147
Caledonia	SWW	*w*	*>2*	*w*	*N*	*b*	*b*	*b*	*b*	*a*	*a*	*a*	*b*	145	142	136	147
Charlie	SRW	*w*	*>2*	*w*	*N*	*b*	*a.1*	*b*	*a*	*a*	*a*	*a*	*b*	142	139	133	146
Coker 9553	SRW	*w*	*>2*	*w*	*N*	*b*	*b*	*b*	*a*	*a*	*a*	*a*	*b*	140	140	131	143
Coker 9804	SRW	*w*	*>2*	*w*	*N*	*b*	*b*	*b*	*a*	*a*	*a*	*a*	*b*	144	140	133	143
Coker 9835	SRW	*w*	*>2*	*w*	*het*	*b*	*het*	*a-CS/a-S64 het*	*a*	*a*	*a*	*a*	*b*	143	140	134	145
Croplan 9101	SRW	*w*	*2/>2 het*	*w*	*N*	*b*	*a.1*	*b*	*a*	*a*	*a*	*b*	*a*	143	140	132	147
D8006W	SWW	*w*	*>2*	*w*	*N*	*b*	*b*	*b*	*a*	*a*	*b*	*a*	*b*	143	140	133	145
Dowell	SRW	*w*	*>2*	*w*	*N*	*b*	*b*	*a-CS*	*a*	*a*	*a*	*a*	*b*	142	141	132	145
E6012	SWW	*w*	*>2*	*w*	*N*	*b*	*het*	*a-CS*	*a/b-Nor het*	*a*	*het*	*a*	*het*	143	139	134	144
Ernie	SRW	*w*	*2*	*w*	*N*	*b*	*a.1*	*b*	*b-Nor*	*a*	*a*	*a*	*a*	-	139	134	-
Evans	SRW	*w*	*>2*	*w*	*N*	*b*	*b*	*b*	*a*	*a*	*b*	*a*	*b*	-	140	135	147
F0014	SWW	*w*	*>2*	*w*	*N*	*b*	*b*	*b*	*a*	*a*	*a*	*a*	*b*	144	141	138	146
F0039	SWW	*w*	*>2*	*w*	*N*	*b*	*b*	*b*	*b*	*a*	*a*	*a*	*b*	145	142	136	147
F0065	SWW	*w*	*>2*	*w*	*N*	*b*	*b*	*b*	*a*	*het*	*het*	*a*	*b*	144	141	136	146
Featherstone 73	SRW	*w*	*>2*	*w*	*N*	*b*	*a.1*	*b*	*b-Nor*	*a*	*a*	*a*	*b*	145	140	136	147
Freedom	SRW	*w*	*>2*	*w*	*N*	*b*	*a.1*	*a-CS*	*b-Nor*	*a*	*a*	*b*	*a*	-	142	135	-
Havoc	SRW	*w*	*>2*	*w*	*N*	*b*	*b*	*b*	*a*	*a*	*a*	*a*	*b*	144	140	135	146
Heilman	SRW	*w*	*>2*	*w*	*N*	*b*	*het*	*b*	*a/b het*	*a*	*a*	*b*	*a*	142	140	132	143
Hilliard	SRW	*w*	*>2*	*w*	*N*	*b*	*het*	*a-S64*	*a*	*a*	*a*	*a*	*b*	-	139	133	146

Supplemental Table S1 Continued

		Vrn loci					*Ppd* loci			*Eps* loci		*Rht* loci		Heading date (day of the year)			
Sample Name	Class	*Vrn-A1*	*vrn-A1* CNV	*Vrn-B1*	*vrn-B1*	*vrn-D3*	*A1*	*B1*	*D1*	*TaELF3-B1*	*TaELF3-D1*	*B1*	*D1*	2015	2016	2017	2018
Hopewell	SRW	*w*	*>2*	*w*	*N*	*a*	*b*	*a-S64*	*b*	*a*	*a*	*b*	*a*	144	142	136	146
Hunker	SRW	*w*	*>2*	*w*	*N*	*b*	*b*	*b*	*a*	*a*	*a*	*a*	*b*	144	141	136	146
Jamestown	SRW	*w*	*2*	*w*	*N*	*b*	*a.1*	*b*	*a*	*a*	*b*	*a*	*b*	139	139	130	144
Jupiter	SWW	*w*	*>2*	*w*	*N*	*b*	*het*	*b*	*a/b-Nor het*	*a*	*het*	*b*	*a*	-	141	135	-
Kenton	SRW	*w*	*>2*	*w*	*N*	*b*	*a.1*	*b*	*b-Nor*	*a*	*b*	*a*	*a*	145	141	136	146
Key	SRW	*w*	*>2*	*w*	*N*	*a*	*b*	*b*	*b-Nor*	*a*	*a*	*a*	*a*	145	142	136	-
Kristy	SRW	*w*	*>2*	*w*	*null*	*b*	*b*	*b*	*a*	*a*	*a*	*a*	*b*	143	139	133	-
KWS 019	SWW	*w*	*>2*	*w*	*N*	*b*	*b*	*b*	*a*	*het*	*a*	*a*	*b*	144	141	137	148
KWS 023	SRW	*w*	*>2*	*w*	*N*	*b*	*a.1*	*b*	*a/b het*	*a*	*a*	*b*	*a*	144	140	137	145
KWS 026	SRW	*w*	*>2*	*w*	*N*	*b*	*a.1*	*null*	*a*	*a*	*b*	*b*	*a*	140	140	135	145
KWS 028	SRW	*w*	*>2*	*w*	*N*	*b*	*a.1*	*b*	*b*	*a*	*a*	*b*	*a*	145	142	135	146
KWS 033	SWW	*w*	*>2*	*w*	*N*	*b*	*b*	*b*	*a*	*a*	*b*	*a*	*b*	146	143	131	149
KWS 034	SWW	*w*	*>2*	*w*	*N*	*b*	*a.1*	*b*	*b-Nor*	*a*	*b*	*a*	*b*	147	142	136	149
KWS 036	SWW	*w*	*>2*	*w*	*N*	*b*	*b*	*b*	*a/b het*	*a*	*het*	*a*	*het*	144	141	136	147
KWS 050	SRW	*w*	*>2*	*w*	*N*	*b*	*b*	*null*	*a*	*a*	*a*	*a*	*b*	-	141	135	-
KWS 052	SRW	*w*	*>2*	*w*	*N*	*b*	*b*	*b*	*a*	*a*	*a*	*a*	*b*	-	140	133	-
KWS 053	SRW	*w*	*>2*	*w*	*N*	*b*	*a.1*	*a-CS*	*b-Nor*	*a*	*b*	*a*	*a*	-	139	128	-
L11417	SRW	*w*	*>2*	*w*	*N*	*a*	*a.1*	*b*	*b*	*a*	*a*	*a*	*b*	141	139	132	143
L11418	SRW	*w*	*>2*	*w*	*N*	*a*	*a.1*	*b*	*b-Nor*	*a*	*a*	*a*	*b*	142	140	133	144
L11419	SRW	*w*	*>2*	*w*	*N*	*b*	*a.1*	*b*	*b*	*a*	*a*	*b*	*a*	141	140	133	143
L11420	SRW	*w*	*>2*	*w*	*N*	*b*	*a.1*	*b*	*b*	*a*	*a*	*b*	*a*	142	139	133	142
L11421	SRW	*w*	*>2*	*w*	*N*	*b*	*a.1*	*a-CS*	*b-Nor*	*a*	*b*	*a*	*a*	141	139	133	142

Supplemental Table S1 Continued

		Vrn loci					*Ppd* loci			*Eps* loci		*Rht* loci		Heading date (day of the year)			
Sample Name	Class	*Vrn -A1*	*vrn-A1* CNV	*Vrn-B1*	*vrn-B1*	*vrn-D3*	*A1*	*B1*	*D1*	*TaELF 3-B1*	*TaELF 3-D1*	*B1*	*D1*	2015	2016	2017	2018
L11422	SRW	*w*	*>2*	*w*	*N*	*b*	*a.1*	*a-CS*	*a/b-Nor het*	*a*	*b*	*a*	*het*	140	139	133	142
L11425	SRW	*w*	*>2*	*w*	*N*	*b*	*b*	*b*	*a*	*a*	*a*	*b*	*a*	142	140	134	144
L11428	SRW	*w*	*>2*	*w*	*N*	*b*	*b*	*b*	*a*	*a*	*a*	*b*	*a*	142	139	133	144
L29235	SRW	*w*	*>2*	*w*	*N*	*b*	*het*	*a-S64*	*b*	*a*	*a*	*b*	*a*	143	140	134	146
LA754	SRW	*w*	*2/>2 het*	*w*	*N*	*b*	*het*	*b*	*a*	*a*	*a*	*a*	*b*	143	140	134	144
LA841	SRW	*w*	*2/>2 het*	*w*	*N*	*b*	*a.1*	*b*	*a*	*a*	*a*	*a*	*b*	142	140	132	145
LCS News	SRW	*w*	*>2*	*w*	*null*	*b*	*a.1*	*null*	*b-Nor*	*a*	*b*	*b*	*a*	144	141	135	146
M09L-9513	SRW	*w*	*>2*	*w*	*N*	*b*	*het*	*b*	*a*	*het*	*b*	*b*	*a*	144	140	134	146
M09L-9547	SRW	*w*	*>2*	*w*	*N*	*b*	*a.1*	*b*	*b-Nor*	*a*	*b*	*b*	*a*	142	140	135	146
M10-1100#	SRW	*w*	*>2*	*w*	*N*	*b*	*b*	*b*	*a*	*het*	*a*	*het*	*b*	145	140	136	-
M10-1277	SRW	*w*	*>2*	*w*	*N*	*b*	*b*	*b*	*a*	*a*	*a*	*a*	*b*	142	139	131	141
M11*3144CW	SRW	*w*	*>2*	*w*	*N*	*b*	*b*	*b*	*b*	*a*	*a*	*a*	*b*	-	142	137	148
M11-2024#	SRW	*w*	*>2*	*w*	*N*	*b*	*b*	*b*	*a*	*a*	*b*	*a*	*b*	139	139	131	-
McCormick	SRW	*w*	*>2*	*w*	*N*	*b*	*a.1*	*b*	*b*	*a*	*a*	*a*	*b*	141	140	134	145
Malabar	SRW	*w*	*>2*	*w*	*N*	*a*	*b*	*b*	*b/b-Nor het*	*a*	*a*	*b*	*a*	146	142	139	146
MCIA Venus	SWW	*w*	*>2*	*w*	*N*	*b*	*b*	*b*	*a*	*a*	*a*	*a*	*b*	142	139	131	143
Milton	SRW	*w*	*>2*	*w*	*N*	*b*	*b*	*b*	*a*	*a*	*a*	*a*	*b*	143	141	134	147
Morrin	SRW	*w*	*>2*	*w*	*N*	*b*	*a.1*	*a-S64*	*b-Nor*	*a*	*a*	*b*	*a*	-	141	136	146
Oakes	SRW	*w*	*>2*	*w*	*N*	*b*	*het*	*a-CS*	*a*	*a*	*b*	*a*	*b*	145	140	135	145
Oasis	SRW	*w*	*>2*	*w*	*N*	*b*	*b*	*a-CS*	*b/b-Nor het*	*a*	*het*	*a*	*a*	145	142	137	146
OH02-12686	SRW	*w*	*>2*	*w*	*N*	*b*	*b*	*b*	*b*	*a*	*a*	*b*	*a*	148	143	140	149
OH04-264-58	SRW	*w*	*>2*	*w*	*N*	*b*	*b*	*b*	*a*	*a*	*b*	*a*	*b*	147	142	135	146
Otsego	SRW	*w*	*>2*	*w*	*N*	*b*	*b*	*a-CS*	*b*	*a*	*a*	*a*	*a*	144	141	136	146

Supplemental Table S1 Continued

		Vrn loci					*Ppd* loci			*Eps* loci		*Rht* loci		Heading date (day of the year)			
Sample Name	Class	*Vrn -A1*	*vrn-A1* CNV	*Vrn-B1*	*vrn-B1*	*vrn-D3*	*A1*	*B1*	*D1*	*TaELF 3-B1*	*TaELF 3-D1*	*B1*	*D1*	2015	2016	2017	2018
Pierson	SRW	*w*	*>2*	*w*	*N*	*b*	*b*	*b*	*b*	*a*	*het*	*b*	*a*	142	140	136	146
Pioneer 25R32	SRW	*w*	*>2*	*w*	*N*	*b*	*b*	*b*	*a*	*a*	*a*	*a*	*b*	145	140	133	146
Pioneer 25R34	SRW	*w*	*>2*	*w*	*N*	*b*	*b*	*b*	*a*	*a*	*a*	*a*	*b*	144	140	134	145
Pioneer 25R39	SRW	*w*	*>2*	*w*	*N*	*b*	*b*	*b*	*a*	*a*	*a*	*a*	*b*	143	141	136	145
Pioneer 25R40	SRW	*w*	*>2*	*w*	*N*	*b*	*b*	*b*	*a*	*a*	*a*	*a*	*b*	144	141	134	146
Pioneer 25R46	SRW	*w*	*>2*	*w*	*N*	*b*	*b*	*a-CS*	*a*	*a*	*a*	*a*	*b*	144	140	134	145
Pioneer 25R47	SRW	*w*	*>2*	*w*	*N*	*b*	*b*	*b*	*a*	*a*	*a*	*a*	*b*	144	140	133	144
Pioneer 25R62	SRW	*w*	*>2*	*w*	*N*	*b*	*a.1*	*b*	*a*	*b*	*a*	*a*	*b*	143	140	131	145
Pioneer 25R77	SRW	*w*	*>2*	*w*	*N*	*b*	*het*	*b*	*a*	*a*	*a*	*a*	*b*	143	140	131	145
Pioneer 25W43	SWW	*w*	*>2*	*w*	*N*	*b*	*b*	*b*	*a*	*b*	*a*	*a*	*b*	145	140	134	145
Pioneer 26R10	SRW	*w*	*>2*	*w*	*N*	*b*	*b*	*b*	*a*	*a*	*a*	*a*	*b*	145	140	134	147
Pioneer 26R53	SRW	*w*	*>2*	*w*	*N*	*b*	*b*	*b*	*a*	*a*	*b*	*a*	*b*	143	140	133	146
Pur 04606	SRW	*w*	*>2*	*w*	*N*	*a*	*b*	*b*	*b*	*a*	*b*	*b*	*a*	142	140	135	146
Pur 05222	SRW	*w*	*>2*	*w*	*N*	*b*	*het*	*a-CS*	*a/b het*	*het*	*b*	*b*	*a*	140	140	132	144
Pur 05247	SRW	*w*	*>2*	*w*	*N*	*b*	*a.1*	*b*	*a/b het*	*a*	*a*	*b*	*a*	145	142	137	147
Ricochet	SRW	*w*	*>2*	*w*	*N*	*b*	*a.1*	*a-CS*	*a*	*a*	*a*	*a*	*b*	144	141	134	145
RS907	SRW	*w*	*>2*	*w*	*N*	*b*	*het*	*b*	*a*	*a*	*b*	*a*	*b*	142	141	132	145
RS910	SRW	*w*	*>2*	*w*	*N*	*b*	*b*	*b*	*a*	*a*	*a*	*b*	*a*	144	140	134	146
RS935	SRW	*w*	*>2*	*w*	*N*	*b*	*b*	*b*	*a*	*a*	*a*	*b*	*a*	141	140	131	144
RS967	SRW	*w*	*>2*	*w*	*N*	*b*	*b*	*b*	*a*	*a*	*b*	*b*	*a*	142	140	132	144
RS972	SRW	*w*	*>2*	*w*	*N*	*b*	*b*	*b*	*a*	*a*	*a*	*a*	*b*	144	140	135	146
RSxp511	SRW	*w*	*>2*	*w*	*N*	*b*	*a.1*	*b*	*a*	*a*	*a*	*a*	*b*	-	142	135	146
SC 1315	SRW	*w*	*>2*	*w*	*N*	*b*	*b*	*b*	*a*	*a*	*a*	*b*	*a*	141	140	130	144
Saranac	SWW	*w*	*>2*	*w*	*N*	*b*	*b*	*b*	*b/b-Nor het*	*a*	*a*	*a*	*het*	145	141	135	145

Supplemental Table S1 Continued

		Vrn loci					*Ppd* loci			*Eps* loci		*Rht* loci		Heading date (day of the year)			
Sample Name	Class	*Vrn -A1*	*vrn-A1* CNV	*Vrn-B1*	*vrn-B1*	*vrn-D3*	*A1*	*B1*	*D1*	*TaELF 3-B1*	*TaELF 3-D1*	*B1*	*D1*	2015	2016	2017	2018
SC 1325	SRW	*w*	*>2*	*w*	*N*	*b*	*a.1*	*b*	*a*	*a*	*a*	*b*	*a*	142	140	131	142
SC 1335	SRW	*w*	*>2*	*w*	*N*	*b*	*b*	*b*	*a*	*a*	*a*	*b*	*a*	143	140	134	145
SC-Exp102	SRW	*w*	*>2*	*w*	*N*	*b*	*b*	*b*	*a*	*a*	*a*	*a*	*b*	-	140	130	145
SC-Exp142	SRW	*w*	*>2*	*w*	*N*	*b*	*b*	*b*	*a*	*b*	*a*	*b*	*a*	-	140	131	143
SC 13S26	SRW	*w*	*>2*	*w*	*N*	*b*	*b*	*b*	*a*	*a*	*a*	*b*	*a*	-	140	134	144
Sebree	SRW	*w*	*>2*	*w*	*N*	*a*	*het*	*a-CS*	*a/b het*	*a*	*het*	*b*	*a*	139	139	127	139
Shirley	SRW	*w*	*>2*	*w*	*N*	*b*	*a.1*	*null*	*a*	*a*	*a*	*b*	*a*	145	140	134	147
Sisson	SRW	*w*	*>2*	*w*	*N*	*b*	*a.1*	*a-CS*	*b-Nor*	*a*	*a*	*a*	*b*	-	140	132	-
Southern Harvest 3200	SRW	*w*	*>2*	*w*	*N*	*b*	*a.1*	*b*	*b-Nor*	*a*	*a*	*a*	*b*	143	140	136	146
SR 5111	SRW	*w*	*>2*	*w*	*N*	*b*	*b*	*b*	*a*	*a*	*b*	*a*	*b*	-	139	130	142
SS 8412	SRW	*w*	*>2*	*w*	*N*	*b*	*a.1*	*b*	*b-Nor*	*het*	*a*	*a*	*b*	143	140	135	147
SS 8415	SRW	*w*	*>2*	*w*	*N*	*b*	*b*	*b*	*a*	*a*	*a*	*a*	*b*	142	140	134	146
Sunburst	SRW	*w*	*>2*	*w*	*N*	*b*	*b*	*b*	*a*	*a*	*a*	*a*	*b*	145	140	135	146
SY 100	SRW	*w*	*>2*	*w*	*N*	*b*	*b*	*b*	*a*	*a*	*a*	*a*	*b*	-	139	135	147
SY 474	SRW	*w*	*>2*	*w*	*N*	*b*	*het*	*b*	*a/b-Nor het*	*a*	*b*	*b*	*a*	144	141	135	146
SY 483	SRW	*w*	*>2*	*w*	*N*	*b*	*b*	*b*	*a*	*a*	*a*	*b*	*a*	145	142	136	148
SY Cypress	SRW	*w*	*2/>2 het*	*w*	*het*	*b*	*het*	*a-CS/a-S64 het*	*a/b het*	*a*	*a*	*a*	*b*	141	139	132	144
SY Harrison	SRW	*w*	*>2*	*w*	*N*	*b*	*b*	*b*	*a*	*a*	*a*	*a*	*b*	142	140	133	146
Terral TV 8861	SRW	*w*	*>2*	*w*	*N*	*b*	*b*	*b*	*a*	*a*	*a*	*a*	*b*	145	141	136	147
TN 1201	SRW	*w*	*>2*	*w*	*N*	*a*	*a.1*	*b*	*b-Nor*	*a*	*a*	*a*	*b*	142	140	134	145
TN 1202	SRW	*w*	*>2*	*w*	*het*	*b*	*a.1*	*b*	*b*	*a*	*a*	*a*	*b*	145	141	136	147
Tribute	SRW	*w*	*>2*	*w*	*N*	*b*	*a.1*	*b*	*b-Nor*	*a*	*het*	*a*	*b*	142	139	134	145
USG 3201	SRW	*w*	*>2*	*w*	*N*	*b*	*b*	*b*	*a*	*a*	*a*	*a*	*b*	141	139	132	144

Supplemental Table S1 Continued

		Vrn loci					*Ppd* loci			*Eps* loci		*Rht* loci		Heading date (day of the year)			
Sample Name	Class	*Vrn -A1*	*vrn-A1* CNV	*Vrn-B1*	*vrn-B1*	*vrn-D3*	*A1*	*B1*	*D1*	*TaELF 3-B1*	*TaELF 3-D1*	*B1*	*D1*	2015	2016	2017	2018
USG 3251	SRW	*w*	*>2*	*w*	*N*	*b*	*b*	*b*	*a*	*a*	*a*	*a*	*b*	143	141	134	147
USG 3315	SRW	*w*	*>2*	*w*	*N*	*b*	*a.1*	*b*	*a*	*a*	*b*	*a*	*b*	143	139	134	147
USG 3555	SRW	*w*	*>2*	*w*	*N*	*b*	*b*	*null*	*a*	*a*	*a*	*a*	*b*	144	139	133	146
VA05W-151	SRW	*w*	*>2*	*w*	*N*	*b*	*a.1*	*b*	*b*	*a*	*het*	*het*	*het*	141	140	135	145
VA05W-251	SRW	*w*	*1/>2 het*	*w*	*N*	*b*	*b*	*a-CS*	*a*	*a*	*a*	*a*	*b*	145	140	136	145
VA08MAS-369	SRW	*w*	*1*	*w*	*N*	*a*	*a.1*	*b*	*b*	*a*	*a*	*a*	*b*	143	139	135	146
VA10W-119	SRW	*w*	*>2*	*w*	*N*	*b*	*b*	*b*	*a*	*a*	*a*	*a*	*b*	142	139	133	145
VA10W-96	SRW	*w*	*>2*	*w*	*N*	*b*	*a.1*	*a-S64*	*a*	*a*	*a*	*a*	*b*	138	139	127	140
VA10W-123	SRW	*w*	*>2*	*w*	*N*	*b*	*b*	*a-S64*	*a*	*a*	*a*	*a*	*b*	139	139	131	144
VA11W-106	SRW	*w*	*>2*	*w*	*N*	*b*	*b*	*b*	*a*	*a*	*a*	*a*	*b*	146	140	134	145
VA11W-279	SRW	*w*	*>2*	*w*	*N*	*b*	*b*	*null*	*a*	*a*	*a*	*a*	*b*	-	139	134	145
VA11W-313	SRW	*w*	*>2*	*w*	*N*	*b*	*b*	*a-S64*	*a*	*a*	*a*	*a*	*b*	-	139	131	143
VA12W-72	SRW	*w*	*>2*	*w*	*N*	*b*	*b*	*a-S64*	*a*	*a*	*a*	*a*	*b*	-	139	131	144
Vandal	SRW	*w*	*>2*	*w*	*N*	*b*	*b*	*b*	*a*	*a*	*a*	*a*	*b*	144	141	135	147
W1104	SRW	*w*	*>2*	*w*	*N*	*b*	*b*	*b*	*a*	*b*	*a*	*b*	*a*	143	141	135	146
WB-196	SRW	*w*	*>2*	*w*	*N*	*b*	*a.1*	*b*	*b-Nor*	*a*	*a*	*a*	*b*	144	139	136	147
Yorktown	SRW	*w*	*>2*	*w*	*N*	*b*	*het*	*a-S64*	*a/b het*	*a*	*a*	*het*	*het*	143	141	133	144
Korean winter wheat																	
Alchan	HRW	*w*	*>2*	*w*	*N*	*b*	*b*	*b*	*a*	*a*	*a*	*a*	*b*	-	-	119	134
Anbaek	HRW	*w*	*1*	*w*	*N*	*b*	*b*	*b*	*a*	*a*	*a*	*a*	*a*	-	-	119	134
Baekjoong	HWW	*w*	*1*	*w*	*N*	*a*	*b*	*b*	*a*	*a*	*a*	*a*	*b*	-	-	116	135
Chungkye	SRW	*w*	*1*	*w*	*N*	*b*	*b*	*b*	*a*	*a*	*a*	*a*	*b*	-	-	119	135
Dahong	SRW	*w*	*1*	*w*	*N*	*b*	*b*	*a-CS*	*a*	*a*	*a*	*a*	*b*	-	-	119	134
Dajoong	HRW	*w*	*1*	*w*	*N*	*b*	*b*	*b*	*a*	*a*	*a*	*a*	*a*	-	-	116	135

Supplemental Table S1 Continued

		Vrn loci					*Ppd* loci			*Eps* loci		*Rht* loci		Heading date (day of the year)			
Sample Name	Class	*Vrn-A1*	*vrn-A1* CNV	*Vrn-B1*	*vrn-B1*	*vrn-D3*	*A1*	*B1*	*D1*	*TaELF3-B1*	*TaELF3-D1*	*B1*	*D1*	2015	2016	2017	2018
Eunpa	HRW	*w*	*1*	*w*	*N*	*a*	*b*	*null*	*a*	*a*	*a*	*a*	*b*	-	-	119	135
Geuru	S/HRW	*w*	*1/>2 het*	*w*	*N*	*b*	*b*	*a-S64*	*het*	*a*	*a*	*het*	*b*	-	-	118	135
Gobun	HRW	*w*	*1*	*w*	*N*	*a*	*b*	*b*	*a*	*a*	*a*	*a*	*b*	-	-	117	135
Goso	SRW	*w*	*1*	*w*	*N*	*b*	*b*	*b*	*a*	*a*	*a*	*b*	*a*	-	-	116	132
Hanbaek	HWW	*w*	*1*	*w*	*N*	*b*	*b*	*b*	*a*	*a*	*a*	*a*	*a*	-	-	118	135
Iksan334	SRW	*w*	*2/>2 het*	*w*	*N*	*a*	*b*	*b*	*a*	*a*	*a*	*b*	*a*	-	-	116	132
Jeokjoong	HWW	*w*	*1*	*w*	*N*	*a*	*b*	*b*	*a*	*a*	*a*	*a*	*b*	-	-	114	130
Jinpoom	HRW	*w*	*1*	*w*	*N*	*a*	*b*	*b*	*a*	*a*	*a*	*a*	*b*	-	-	118	135
Joeun	HRW	*w*	*1*	*w*	*N*	*a*	*b*	*b*	*a*	*a*	*a*	*a*	*a*	-	-	114	130
Jokyung	HWW	*w*	*>2*	*w*	*N*	*a*	*b*	*b*	*a*	*a*	*a*	*b*	*a*	-	-	117	132
Jonong	SRW	*w*	*1*	*w*	*N*	*b*	*b*	*b*	*a*	*a*	*a*	*a*	*a*	-	-	113	130
Jopoom	HRW	*w*	*1*	*w*	*N*	*b*	*b*	*b*	*a*	*a*	*a*	*a*	*a*	-	-	113	130
Keumkang	HWW	*w*	*1*	*w*	*N*	*a*	*b*	*b*	*a*	*a*	*a*	*b*	*a*	-	-	117	135
Milsung	SRW	*w*	*1*	*w*	*N*	*b*	*b*	*b*	*a*	*a*	*a*	*b*	*a*	-	-	116	132
Namhae	SRW	*w*	*1*	*w*	*N*	*b*	*b*	*b*	*a*	*a*	*a*	*a*	*b*	-	-	116	132
Olgeuru	SRW	*w*	*1*	*w*	*N*	*b*	*b*	*b*	*a*	*a*	*a*	*b*	*a*	-	-	116	132
Olmil	SRW	*w*	*1*	*w*	*N*	*b*	*b*	*a-CS*	*a*	*a*	*a*	*a*	*b*	-	-	119	136
Saeol	SRW	*w*	*1*	*w*	*N*	*a*	*b*	*b*	*a*	*a*	*a*	*b*	*a*	-	-	114	130
Seodun	HRW	*w*	*1*	*w*	*N*	*a*	*b*	*b*	*a*	*a*	*a*	*a*	*b*	-	-	118	136
Shinmichal	SRW	*w*	*1*	*w*	*N*	*b*	*b*	*b*	*a*	*a*	*a*	*b*	*a*	-	-	119	136
Shinmichall	SRW	*w*	*>2*	*w*	*N*	*b*	*b*	*b*	*a*	*a*	*a*	*b*	*a*	-	-	116	134
Sooan	HRW	*w*	*1*	*w*	*N*	*a*	*b*	*b*	*a*	*a*	*a*	*a*	*a*	-	-	115	130
Sookang	HRW	*w*	*1*	*w*	*N*	*a*	*b*	*b*	*a*	*a*	*a*	*b*	*a*	-	-	117	132
Tapdong	HRW	*w*	*>2*	*w*	*N*	*b*	*b*	*b*	*a*	*a*	*a*	*a*	*b*	-	-	118	135

Supplemental Table S1 Continued

		Vrn loci					*Ppd* loci			*Eps* loci		*Rht* loci		Heading date (day of the year)			
Sample Name	Class	*Vrn-A1*	*vrn-A1* CNV	*Vrn-B1*	*vrn-B1*	*vrn-D3*	*A1*	*B1*	*D1*	*TaELF3-B1*	*TaELF3-D1*	*B1*	*D1*	2015	2016	2017	2018
Uri	SRW	*w*	*1*	*w*	*N*	*a*	*b*	*b*	*a*	*a*	*a*	*a*	*b*	-	-	116	134
Yeonbaek	HWW	*w*	*1*	*w*	*N*	*a*	*b*	*b*	*a*	*a*	*a*	*a*	*b*	-	-	118	133

SRW, soft red winter; SWW, soft white winter; HRW, hard red winter; HWW, hard white winter.

Supplemental Table S2. Analysis of variance of genotype, year and genotype × year for heading date in 121 eastern U.S. soft winetr wheat genotypes

Source	Degree of freedom	Type III SS	Mean Square	*p* value	H^2
Genotype	120	1052.3	8.8	<0.0001	0.86
Year	3	8706.6	2902.2	<0.0001	
Genotype × year	360	445.9	1.2	<0.0001	

Heritability calculated in terms of mean square where $H^2 = (MS_{\text{Genotype}} - MS_{\text{Genotype × Year}}) / MS_{\text{Genotype}}$.

Supplemental Table S3. Correlations between heading dates of 121 eastern U.S. soft winter wheat in different crop years

Heading date	2015	2016	2017
2016	0.62***		
2017	0.73***	0.52***	
2018	0.72***	0.57***	0.73***

***$p < 0.0001$

Supplemental Table S4. Influences of crop year and the vernalization (*Vrn*), photoperiod (*Ppd*), earliness *per se* (*Eps*) and reduced-height (*Rht*) genes on variations in the heading date of 30 Korean winter wheat varieties observed in two crop years.

Source of variation	Degrees of freedom	Mean square	Proportion of total variance (%)	Proportion of genetic variance (%)
Crop year	1	4100.3***	94.7	
Vrn-A1	1	2.8	0.1	3.9
Vrn-B1	0	0.0	0.0	0.0
Vrn-D3	1	9.5	0.2	13.7
Ppd-A1	0	0.0	0.0	0.0
Ppd-B1	2	4.7	0.2	13.4
Ppd-D1	0	0.0	0.0	0.0
Eps-B1	0	0.0	0.0	0.0
Eps-D1	0	0.0	0.0	0.0
Rht-B1	1	3.9	0.1	5.6
Rht-D1	1	44.3***	1.0	63.4
Error	52	3.0	3.6	

The sum of squares was used to calculate the proportion of total variance contributed by crop years and each gene, and the proportion of genetic variance by each gene.
*** significant at $p < 0.001$.

Supplemental Table S5. Contributions of vernalization (*Vrn*), photoperiod (*Ppd*), earliness *per se* (*Eps*) and reduced-height (*Rht*) genes, and their interactions, to the heading date of 86 eastern U.S. soft winter wheat genotypes in each of four crop years

Source of variation	Degrees of freedom	2015		2016		2017		2018	
		Mean Square	Proportion of total variance (%)	Mean Square	Proportion of total variance (%)	Mean Square	Proportion of total variance (%)	Mean Square	Proportion of total variance (%)
Vrn-A1	2	3.5	2.3	0.8	2.2	1.8	1.2	0.3	0.2
Vrn-B1	2	0.5	0.3	0.7	2.0	0.2	0.2	0.2	0.1
Vrn-D3	1	11.9*	4.0	5.9**	8.0	6.3	2.0	6.8	2.7
Ppd-A1	1	9.0	3.0	2.4	3.3	0.5	0.1	2.6	1.0
Ppd-B1	3	6.1	6.1	0.1	0.4	6.4	6.0	3.6	4.3
Ppd-D1	2	13.9*	9.2	3.6**	9.8	37.5***	23.5	13.6**	10.8
Eps-B1	1	5.1	1.7	0.9	1.3	0.6	0.2	0.7	0.3
Eps-D1	1	5.4	1.8	2.3	3.2	1.6	0.5	7.2	2.9
Rht-B1	1	9.7	3.2	0.2	0.2	0.0	0.0	0.9	0.4
Rht-D1	1	0.1	0.0	0.1	0.1	6.5	2.0	0.0	0.0
*Ppd-B1*Ppd-D1*	4	5.6	7.5	1.5	8.1	5.6	7.0	9.4**	15.0
*Ppd-B1*Vrn-D3*	1	0.5	0.2	0.8	1.2	0.3	0.1	0.3	0.1
*Ppd-D1*Vrn-D3*	1	0.5	0.2	5.8**	7.8	0.3	0.1	0.3	0.1
Error	64	2.9	60.7	0.6	52.4	2.8	57.1	2.4	62.0

Eighty-six eastern US. soft winter wheat varieties with homozygous alleles at each gene were evaluated for four crop years and were included in the model. The sum of squares was used to calculate the proportion of total variance contributed by each gene.

*** significant at $p < 0.001$, ** significant at $p < 0.01$, * significant at $p < 0.05$.

Supplemental Table S6. Contributions of vernalization (*Vrn*), photoperiod (*Ppd*), earliness *per se* (*Eps*) and reduced-height (*Rht*) genes to the heading date of 30 Korean winter wheat varieties in each of two crop years.

		2017		2018	
Source of variation	Degrees of freedom	Mean Square	Proportion of total variance (%)	Mean Square	Proportion of total variance (%)
Vrn-A1	1	2.4	2.3	0.7	0.5
Vrn-B1	0	0.0	0.0	0.0	0.0
Vrn-D3	1	4.2	4.1	5.4	4.3
Ppd-A1	0	0.0	0.0	0.0	0.0
Ppd-B1	2	3.9	7.6	1.2	1.9
Ppd-D1	0	0.0	0.0	0.0	0.0
Eps-B1	0	0.0	0.0	0.0	0.0
Eps-D1	0	0.0	0.0	0.0	0.0
Rht-B1	1	1.1	1.1	3.1	2.4
Rht-D1	1	21.4*	21.0	22.9*	18.2
Error	23	2.8	63.8	4.0	72.7

The sum of squares was used to calculate the proportion of total variance contributed by each gene.
* significant at $p < 0.05$.

Supplemental Table S7. Primer sequences of KASP markers used in this study

Gene	Allele	Marker ID	Primer name	Primer sequence (5′-3′)
Vrn-A1	*Vrn-A1a*	wMAS000033	Vrn-A1_9K0001_AL2	GAGTTTTCCAAAAAGATAGATCAATGTAAAC
			Vrn-A1_9K0001_AL1	AGAGTTTTCCAAAAAGATAGATCAATGTAAAT
			Vrn-A1_9K0001_C1	GTTAGTAGTGATGGTCCAATAATGCCAAA
	Vrn-A1b	wMAS000035	Vrn-A1b-Marq_AL2	GTTTTGGCCTGGCCATCCTCA
			Vrn-A1b-Marq_AL1	GTTTTGGCCTGGCCATCCTCC
			Vrn-A1b-Marq_C1	TATCAGGTGGTTGGGTGAGGACGT
	vrn-A1, CNV[a]>2	vrn-A1exon4	Vrn-A1_Exon4_F1	AGGCATCTCATGGGAGAGGATC
			Vrn-A1_Exon4_F2	CAGGCATCTCATGGGAGAGGATT
			Vrn-A1_Exon4_R	CCAGTTGCTGCAACTCCTTGAGATT
	vrn-A1, CNV=2	vrn-A1exon7	Vrn-A1_Exon7_F1	TGAGTTTGATCTTGCTGCGCCG
	vrn-A1, CNV=1	vrn-A1exon7	Vrn-A1_Exon7_F2	CTGAGTTTGATCTTGCTGCGCCA
			Vrn-A1_Exon4_R	CTTCCCCACAGCTCGTGGAGAA
Vrn-B1	*Vrn-B1a*	Vrn-B1_I_D	Vrn-B1_D_A2	GGCAGCTAATGTGGGGTAGTCT
			Vrn-B1_D_C1s	ATTCGTATTGCTAGCTCCGGCCAT
			Vrn-B1_I_ALG	CAACCTCCACGGTTTCAAAAAGTAG
			Vrn-B1_I_C1	ATATTTACTAAGCAGCGGTCATTCCGAT
	Vrn-B1b	wMAS000037	Vrn-B1_B_ALC	GCGCAAGCGGGAGCTACATC
			Vrn-B1_B_ALG	TGCGCAAGCGGGAGCTACATG
			Vrn-B1_B_C1	GCCATGAACAACAAAGGGGGTGGT
	Vrn-B1c	Vrn-B1_C	Vrn-B1_C _ALT	CCTAAACAGGGGCAGAACACTA
			Vrn-B1_C _ALG	CCTAAACAGGGGCAGAACACTG
			Vrn-B1_C _C	GACCCCAGGGCCTATGAATGTAATT
	vrn-B1-Neuse	TaVrn-B1_1752	TaVrnB1_1752_AF2	GGAATGACCGCTGCTTAGTAAATATA
	vrn-B1-AGS2000	TaVrn-B1_1752	TaVrnB1_1752_CF1	GGAATGACCGCTGCTTAGTAAATATC

			TaVrnB1_1752_R	GATTTAGCACCTCAACATACAGGTCT
Vrn-D3	*vrn-D3a*	VRN-D3-F6/VRN-D3-R8	VRN-D3-F6	CTTCTATTCACATGTTTCGTTCATG
	vrn-D3b	VRN-D3-F6/VRN-D3-R8	VRN-D3-R8	ACGAGCACGAAGCGATGGATCGC
Ppd-A1	*Ppd-A1a.1*	Ppd-A1prodel	Ppd-A1prodel_AL2	GCGGCGAGCCGGTTAATCG
	Ppd-A1b	Ppd-A1prodel	Ppd-A1prodel_AL1	TTTCGGTGTTTGACTTCAGGCG
			Ppd-A1prodel_C1	GTGGCGTACTCCCTCCGTTTCTT
Ppd-B1	*Ppd-B1a-Chinese Spring*	wMAS000027	TaPpdBJ001tR	GACGTTATGAACGCTTGGCA
			TaPpdBJ001iR	CCGTTTTCGCGGCCTT
			TaPpdBJ001tF	GGGTTCGTCGGGAGCTGT
	Ppd-B1a-Sonora 64	TaPpdBJ003	TaPpdBJ003F	CGTGAAGAGCTAGCGATGAACA
			TaPpdBJ003R	TGGGCACGTTAACACACCTTT
	Ppd-B1b	wMAS000027	TaPpdBJ001tR	GACGTTATGAACGCTTGGCA
			TaPpdBJ001iR	CCGTTTTCGCGGCCTT
			TaPpdBJ001tF	GGGTTCGTCGGGAGCTGT
Ppd-D1	*Ppd-D1a*	wMAS000024	TaPpdDD001RI	CAAGGAAGTATGAGCAGCGGTT
	Ppd-D1b	wMAS000024	TaPpdDD001RD	AAGAGGAAACATGTTGGGGTCC
			TaPpdDD001FL	GCCTCCCACTACACTGGGC
	Ppd-D1b-Norstar	TaPpdDD002	TaPpdDD002FL	GGTCTCCAATCAAGGCGGT
			TaPpdDD002RI	AAGGTGACCAAGTTCATGCTCGAGCAGCTCCCGACG
			TaPpdDD002RD	GAAGGTCGGAGTCAACGGATTGGGCGAGCAGCTCCAAC
Eps-B1	*TaELF3-B1a*	TaELF3-B1 Kasp	aELF3-B1_Kasp_F	CCCTTGCAGCTCGCT
	TaELF3-B1b	TaELF3-B1 Kasp	TaELF3-B1 Kasp_V	CCCTTGCAGCTCGCC
			TaELF3-B1 Kasp2_generic	CGACCCAACACTCACG

Eps-D1	*TaELF3-D1a*	TaELF3-D1 Kasp2	TaELF3-D1_ Kasp2_F	GCCTCAGAATCAGTGGCTT
	TaELF3-D1b	TaELF3-D1 Kasp2	TaELF3-D1_Kasp2_V	GCCTCAGAATCAGTGGCTC
			TaELF3-D1_Kasp2_generic	GTAGACGAACCCTTCCGA
Rht-B1	*Rht-B1a*	wMAS000001	RhtB1_SNP-AL1	CCCATGGCCATCTCSAGCTG
	Rht-B1b	wMAS000001	RhtB1_SNP-AL2	CCCATGGCCATCTCSAGCTA
			RhtB1_SNP-C	TCGGGTACAAGGTGCGGGCG
Rht-D1	*Rht-D1a*	wMAS000002	RhtD1_AL1	CATGGCCATCTCGAGCTRCTC
	Rht-D1b	wMAS000002	RhtD1_AL2	CATGGCCATCTCGAGCTRCTA
			RhtD1_C1	CGGGTACAAGGTGCGCGCC

[a] CNV, copy number variation.

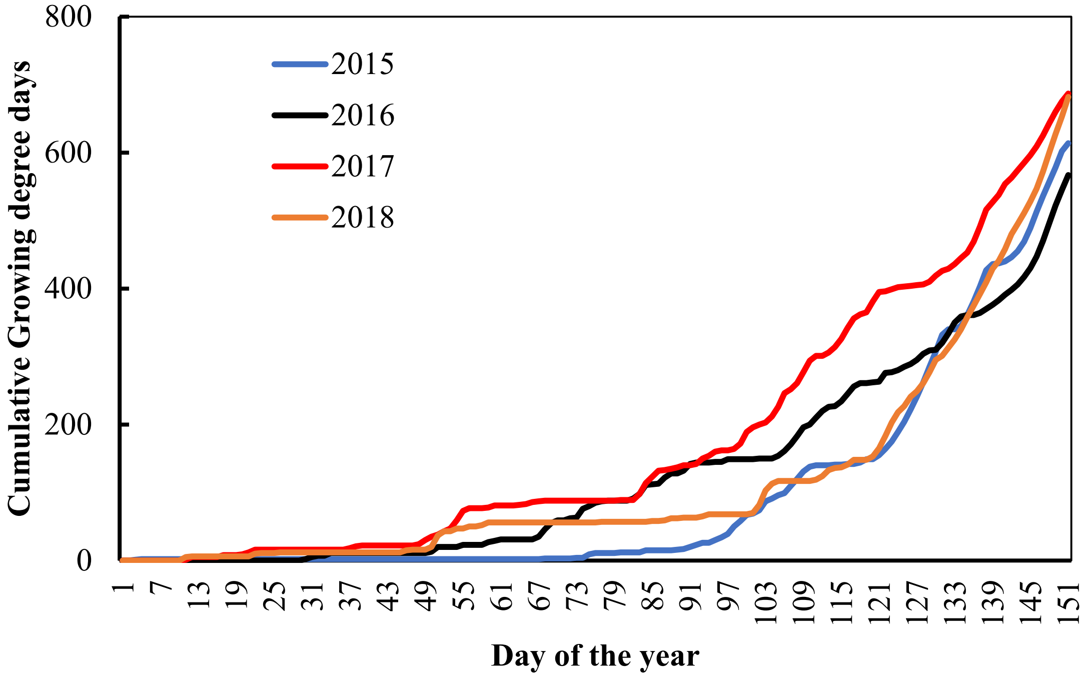

Supplemental Fig S1. Cumulative growing degree days from January 1st to May 31st in Wooster, Ohio, in 2015-2018. Growing degree days = mean daily temperature - 50°F.

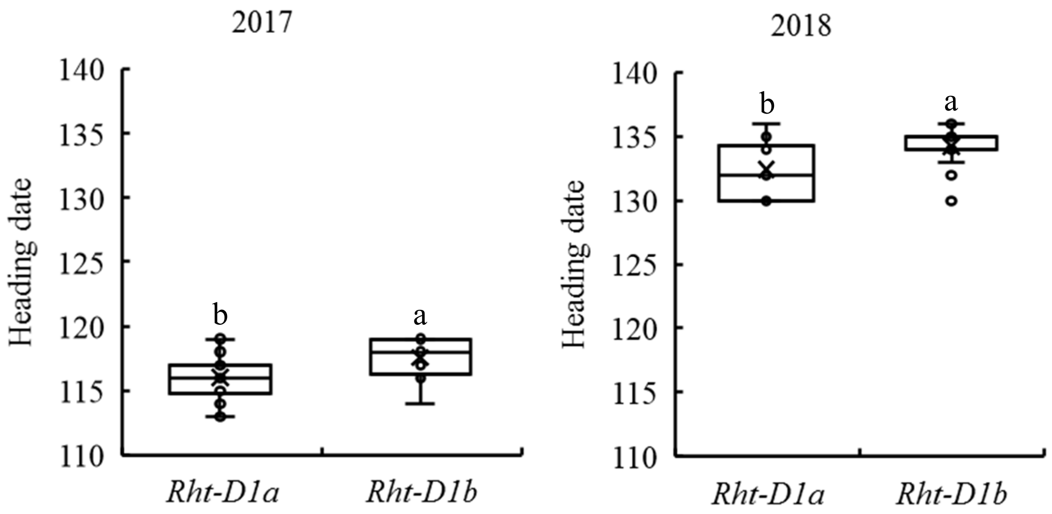

Supplemental Fig. S2. Heading dates of 30 Korean winter wheat varieties with different alleles at the *Rht-D1* gene grown in Wooster, Ohio, in two different years. x indicates the mean. Means with different letters are significantly different at the 0.05 probability level.
